# Integrated RNA-seq and RT-qPCR Workflow Identifies Non-IGH Fusion Transcripts as Individualized Molecular Markers for Monitoring Multiple Myeloma

**DOI:** 10.3390/biomedicines14020354

**Published:** 2026-02-03

**Authors:** Yifei Ren, Yang Lu, Dan Huang, Xuehong Zhang, Beibei Gao, Xijia Wang, Xiangjie Kui, Hongchen Liu, Jiacheng Lou, Jinsong Yan

**Affiliations:** 1 Department of Hematology, Liaoning Medical Center for Hematopoietic Stem Cell Transplantation, The Second Hospital of Dalian Medical University, Dalian 116027, China; 2 Liaoning Key Laboratory of Hematopoietic Stem Cell Transplantation and Translational Medicine, Blood Stem Cell Transplantation Institute, Diamond Bay Institute of Hematology, The Second Hospital of Dalian Medical University, Dalian 116027, China; 3 Department of Pediatrics, Pediatric Oncology and Hematology Center, The Second Hospital of Dalian Medical University, Dalian 116027, China; 4 Department of Hematology, Chaoyang Central Hospital, Chaoyang City 122000, China; 5 Center of Genome and Personalized Medicine, Institute of Cancer Stem Cell, Dalian Medical University, Dalian 116044, China; 6 Department of Pediatrics, The Second Hospital of Dalian Medical University, Dalian 116027, China

**Keywords:** multiple myeloma, fusion genes, minimal residual disease, molecular biomarkers

## Abstract

**Background**: Multiple myeloma (MM) is a hematologic malignancy characterized by clonal plasma cell expansion and diverse genomic rearrangements, including immunoglobulin heavy chain (IGH) translocations. Although RNA sequencing enables the comprehensive detection of IGH-associated fusions, routine molecular monitoring remains limited, particularly in non-secretory MM (NSMM), which lacks measurable serologic markers. **Methods:** Here, we contracted an integrated system combining RNA sequencing (RNA-seq) and reverse transcription quantitative polymerase chain reaction (RT-qPCR) to identify and validate fusion gene-based molecular markers for minimal residual disease (MRD) monitoring. **Results:** The global fusion landscape was delineated by the sequencing analysis of bone marrow samples from 22 newly diagnosed patients with MM. A total of 362 fusion events were identified, of which 190 non-immunoglobulin fusions were selected for detailed characterization. Recurrent breakpoints were concentrated on chromosomes 1 and 19, and five recurrent fusions, *DDX5::EEF1A1*, *OAZ1::KLF2*, *OAZ1::KLF16*, *PFKFB3::LINC02649*, and *PLXNB2::SCO2*, were detected across nine patients. Functional enrichment analyses indicated the significant involvement of these genes in RNA splicing regulation, transcriptional misregulation in cancer-related pathways, and focal adhesion processes. Twenty-three fusion transcripts were validated using RT-PCR and Sanger sequencing, demonstrating high specificity for MM. Longitudinal monitoring revealed that the quantitative assessment of fusion transcript levels enabled earlier relapse detection than flow cytometry, including in NSMM, where conventional MRD tools are ineffective. **Conclusions:** These findings suggest that individualized fusion transcripts serve as robust molecular markers for MRD surveillance. The proposed RNA-seq–RT-qPCR pipeline offers a clinically practical strategy to enhance precision diagnosis and personalized treatment in MM.

## 1. Introduction

Multiple myeloma (MM) is a hematological lymphoid malignancy characterized by the clonal expansion of plasma cells in bone marrow (BM), leading to the overproduction of monoclonal immunoglobulin or its fragments (M protein) [1]. This disease exhibits profound biological heterogeneity, manifested by complex subclonal architecture, diverse driver mutations, and distinct patterns of clonal evolution among patients [2,3]. Traditionally, MM is stratified into hyperdiploid and non-hyperdiploid subtypes. The latter is frequently characterized by immunoglobulin heavy chain (IGH) locus translocations [3,4], most notably t (11;14) (~20% of cases), t (4;14) (~15–20%), and the less common t (14;16) and t (14;20) (~2% each) [2]. These chromosomal rearrangements typically dysregulate partner genes, such as *FGFR3*, *CCND1*, *MYC*, *c-MAF*, and *MAFB*, thereby promoting unchecked proliferation (e.g., *CCND1*), the constitutive activation of growth signaling pathways (*KRAS*/*NRAS*/*BRAF*/*FGFR3*), and transcriptional network reprogramming (*MYC*/*c-MAF*/*MAFB*), which collectively drive tumorigenesis. Collectively, these mechanisms facilitate tumor cell survival, BM homing/adhesion, and drug resistance [5,6,7]. Although RNA sequencing (RNA-seq) allows for the comprehensive detection of IGH translocation-associated fusion transcripts [8], the lack of an analytically detectable, cost-effective, and clinically practical approach for their routine monitoring remains a major limitation in current MM disease assessment.

The diagnosis and monitoring of non-secretory multiple myeloma (NSMM) lack standard disease-specific serology markers, and NSMM frequently presents without targetable IGH fusions, accounting for only 1% to 3% cases [9]. The diagnosis and monitoring of NSMM remain a significant clinical challenge. Current monitoring strategies for NSMM are limited to imaging modalities (PET/CT) and bone marrow-based multi-parameter flow cytometry (FCM) and morphologic assessment [10,11]. In the broader context of MM, minimal residual disease (MRD) is now considered a key independent predictor of progression-free survival and overall survival in newly diagnosed patients [12]. Recently, various types of leukemia have benefited from standardized, highly detectable, and highly specific molecular detection methods, including next-generation FCM (NGF; analytical detectability ≤ 10^−5^), reverse transcription quantitative polymerase chain reaction (RT-qPCR; analytical detectability ≤ 10^−6^), and next-generation sequencing (NGS) [9]. However, the diagnosis and surveillance of MM do not yet benefit from these advantages. Current MRD assessment in MM still relies heavily on fluorescence in situ hybridization, DNA ploidy analysis, FCM, and immunoglobulin gene rearrangement analysis [13], highlighting a substantial need for a comprehensive molecular tracking strategy. Therefore, there is a critical need to develop a universal, user-friendly, and highly detectability molecular marker for patients with MM.

In the present study, a clinically viable workflow was established by combining RNA-seq with RT-qPCR for MRD detection. Using a cohort of 22 patients with newly diagnosed MM who underwent RNA-seq, the global fusion gene landscape of disease was systematically characterized. Beyond canonical IGH translocations, a spectrum of MM-specific fusion transcripts was identified. These novel markers demonstrate exceptional analytical detectability and specificity for molecular MRD monitoring and offer a new class of biomarkers and a refined strategy for precision diagnosis and treatment in MM.

## 2. Materials and Methods

### 2.1. Study Approval and Specimen Selection

A total of 22 patients with newly diagnosed MM were enrolled between April 2019 and October 2021. All BM and oral epithelium samples were collected at diagnosis at the Department of Hematology of the Second Hospital of Dalian Medical University. Ethical approval for this study was granted by the Research Ethics Board of the Second Hospital of Dalian Medical University. The study complied with the Declaration of Helsinki, and written informed consent was obtained from all participants prior to their enrollment.

### 2.2. RNA Sequencing

Total RNA was extracted from plasma cells, which were isolated using a commercial CD138-positive magnetic-activated cell sorting (MACS) kit (Cat. 17877; STEMCELL Technologies, Cambridge, MA, USA). RNA concentration and purity were measured using a Bioanalyzer 2100 (Agilent Technologies, Santa Clara, CA, USA) and a Qubit 2.0 Fluorometer (Life Technologies, Carlsbad, CA, USA). Sequencing libraries were created according to the instructions provided with the TruSeq RNA/TruSeq DNA Sample Preparation Kit (Illumina, San Diego, CA, USA). Under the quality control of the libraries, whole-transcriptome sequencing (WTS) was conducted on a NovaSeq6000 platform (Novogene, Beijing, China) with a paired-end read length of 150 bp.

### 2.3. Sequencing Data Analysis

STAR-Fusion software (version 1.7.0) was used to predict fusion gene transcripts in patients with MM. For BM data, STAR (version 2.7.6a) [14] was used to align WTS reads to the reference genome (hg38) based on transcript coordinates, using a gene annotation format file from GENCODE (Release 27, GRCh38). Statistical over-representation and gene set enrichment were considered significant at nominal *p*-values ≤ 0.01.

### 2.4. Reverse Transcription and Quantitative PCR

Following the extraction of total RNA from the patient samples, reverse transcription was performed using the HiScript II Q RT SuperMix kit (Cat. R223; Vazyme, Nanjing, China), referring to the manufacturer’s protocol. Quantitative PCR was conducted on an ABI-7500 Real-Time PCR system with ChamQ Universal SYBR qPCR Master Mix (Cat. Q711; Vazyme, Nanjing, China). Relative expression of target genes was determined by the 2^−ΔΔCt^ method, normalized to *ABL* or *ACTIN*, with the primers listed in Table 1 and Table S1.

### 2.5. Sanger Sequencing

Amplicons of fusion transcripts obtained by RT-PCR were subjected to Sanger sequencing to verify the predicted fusion junction, as provided in the contig sequence of the Manta fusion output. Sequencing was performed using the BigDye Terminator V.3.1 Sequencing Kit (Applied Biosystems, Foster City, CA, USA).

## 3. Results

### 3.1. Clinical Characteristics

**Figure 1:**
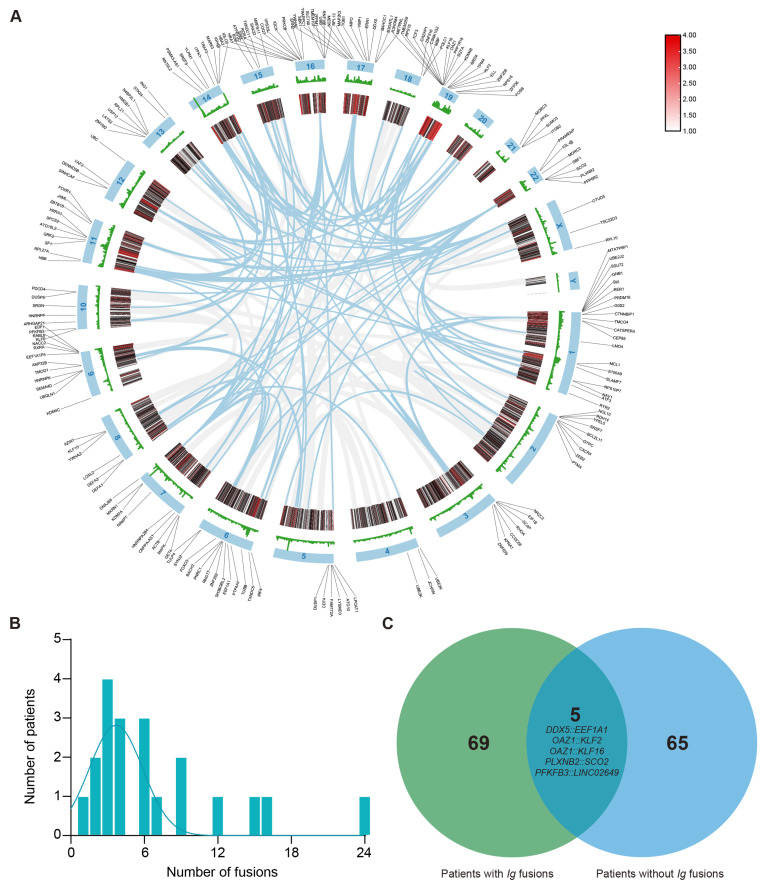
Distribution and characteristics of non-*Ig* fusion events in multiple myeloma. (**A**) The circus plot illustrates the chromosomal distribution of genes with breakpoints. (**B**) Number of fusions among patients who have at least one fusion (average = 7), with all fusions involving immunoglobulin (*Ig*) gene rearrangements excluded. (**C**) Venn diagram indicates that the patients with *Ig* fusion have significantly more fusions than those with non-*Ig* fusion, and fusions of *DDX5::EEF1A1*, *OAZ1::KLF2*, *OAZ1::KLF16*, *PLXNB2::SCO2*, and *PFKFB3::LINC02649* are shown as overlapping recurrent fusions.

A total of 22 patients with newly diagnosed MM were included in this study, combining NGS data with clinical information to generate a comprehensive landscape of fusion events (Figure 1). The patient cohort included 15 men and 7 women, with a median age of 57 years (range, 42–72) (Table 2). Patients were unevenly distributed across Durie–Salmon stages, with 4.5% in stage I (1/22), 13.6% in stage II (3/22), and 81.8% in stage III (18/22). According to the International Staging System, 5 patients (22.7%) were classified as stage I, 4 (18.2%) as stage II, and 13 (59.1%) as stage III. According to the Revised International Staging System, 3 patients (13.6%) were classified as stage I, 7 (31.8%) as stage II, and 12 (54.5%) as stage III. Isotypes were classified as follows: immunoglobulin (Ig)G in nine patients, IgA in five patients, IgD in one patient, light chain in five patients, biclonal in one patient, and non-secretory type in one patient.

### 3.2. Distribution and Characteristics of Non-Ig Fusion Events in Multiple Myeloma

Because IGH fusion genes exhibit highly variable and complex sequences, their potential utility as PCR-based molecular markers is limited. To detect fusion genes with stable junction sequences in the samples of patients with MM, all fusions involving IG gene rearrangements were excluded. Of the 362 fusion genes initially identified, 190 were selected for further analysis. As shown in Figure 1A, these fusion events mapped to all chromosomes except chromosome 20 and chromosome Y. Notably, the prevalence of recurrent breakpoint genes was relatively high on chromosome 1 (*n* = 20, 10.5%) and chromosome 19 (*n* = 19, 10%). Other chromosomes containing ≥ 10 breakpoint genes included chromosome 6 (*n* = 13, 6.8%), chromosome 9 (*n* = 11, 5.8%), chromosome 11 (*n* = 10, 5.3%), chromosome 16 (*n* = 15, 7.9%), and chromosome 17 (*n* = 13, 6.8%). Next, at least one fusion transcript was identifiable in all patients. The number of fusion events per individual ranged from 0 to 24, with an average number of 7 fusions per sample. In particular, four patients (18.2% of patients) had more than 10 fusion events (Figure 1B). Additionally, patients with immunoglobulin rearrangements carried a higher number of fusion genes than patients without such rearrangements (Figure 1C). Among these genes, only five recurrent fusion genes across nine distinct patients were identified in this cohort, namely, *DDX5::EEF1A1*, *OAZ1::KLF2*, *OAZ1::KLF16*, *PLXNB2::SCO2*, and *PFKFB3::LINC02649*. These findings demonstrate that these fusion events are highly individualized and patient-specific.

### 3.3. Functional Consequences of Fusion Gene Breakpoints and Their Biological Implications in MM

**Figure 2:**
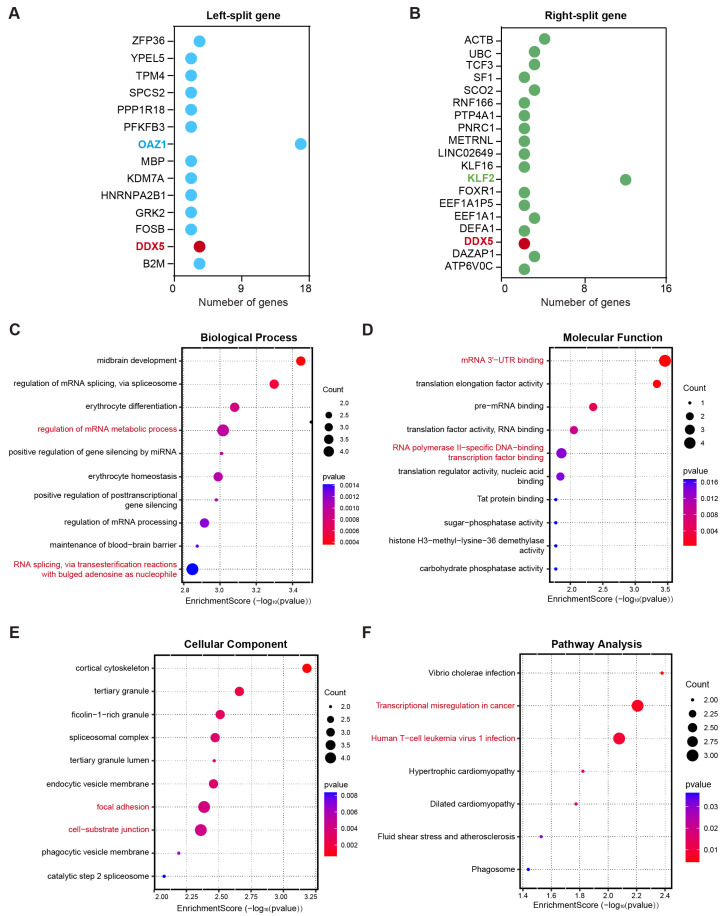
Functional consequences of fusion gene breakpoints and their biological implications in multiple myeloma. (**A**,**B**) The gene sets were involved in left-side breakpoints (**A**) and right-side breakpoints (**B**). (**C**–**E**) Gene Ontology (GO) analysis was performed to annotate the biological processes (**C**), molecular functions (**D**), and cellular components (**E**) associated with these genes that underwent breakage events. (**F**) KEGG pathway analysis demonstrated that the gene sets were markedly enriched in the transcriptional misregulation in cancer pathway and human T-cell leukemia virus 1 infection pathway.

Alteration in gene function caused by fusion gene breakpoints is an important driving force in disease development [15]. To elucidate the potential biological implications of genes involved in chromosomal breakpoints, Gene Ontology (GO) and Kyoto Encyclopedia of Genes and Genomes (KEGG) pathway enrichment analyses were performed. A total of 14 genes were involved in left-side breakpoints, whereas 19 genes were implicated in right-side breakpoints. Among these, the most frequently observed gene at the left breakpoint was *OAZ1* (37%), whereas *KLF2* (12%) was the most recurrent gene at the right breakpoint. Additionally, *DDX5* was identified among the disrupted genes at both breakpoint regions (Figure 2A,B). GO analysis revealed that these genes were predominantly enriched in specific biological processes and molecular functions (Figure 2C,D). With respected to biological processes, the most significantly enriched terms were the regulation of the mRNA metabolic process and RNA splicing via transesterification reactions with bulged adenosine as the nucleophile (adjusted *p*-value < 0.01, Figure 2C). Regarding molecular functions, these genes were predominantly involved in RNA polymerase II-specific DNA-binding transcription factor binding and mRNA 3′-UTR binding (Figure 2D). For cellular components, significant enrichment was observed in cell–substrate junctions, cortical cytoskeleton, and focal adhesion (Figure 2E). Notably, the KEGG pathway results demonstrated that this gene set was significantly enriched in the transcriptional misregulation in cancer pathway and the human T-cell leukemia virus 1 infection pathway (Figure 2F). These results indicate that fusion genes may contribute to myeloma development by disrupting the normal transcription and post-transcriptional regulatory networks.

### 3.4. Validation of Fusion Gene Specificity and Identification of Potential Molecular Markers in MM

**Figure 3:**
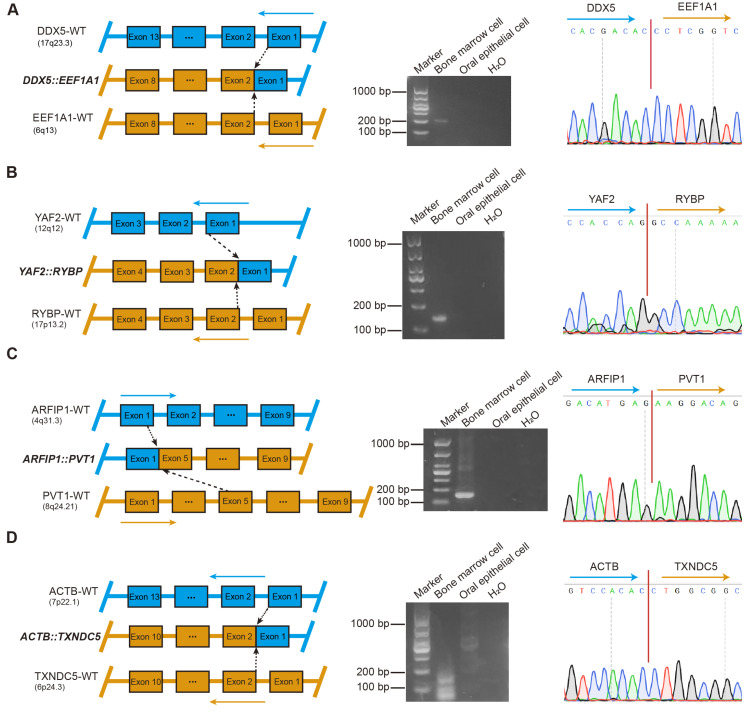
Validation of fusion gene specificity and identification of potential molecular markers in multiple myeloma. (**A**) *DDX5::EEF1A1*. Left: Graphical representation of the formation of *DDX5::EEF1A1* fusion at the chromosome level. Middle: RT-PCR of *DDX5::EEF1A1* from BM cells and oral epithelial cells. ddH_2_O served as the negative control. Marker, size marker. Right: chromatogram from Sanger sequencing of the PCR product, showing the reading frame at the break points. (**B**) *YAF2::RYBP*. Left: Graphical representation of the formation of *YAF2::RYBP* fusion at the chromosome level. Middle: RT-PCR of *YAF2::RYBP* from BM cells and oral epithelial cells. ddH_2_O served as the negative control. Marker, size marker. Right: chromatogram from Sanger sequencing of the PCR product, showing the reading frame at the break points. (**C**) *ARFIP1::PVT1*. Left: Graphical representation of the formation of *ARFIP1::PVT1* fusion at the chromosome level. Middle: RT-PCR of *ARFIP1::PVT1* from BM cells and oral epithelial cells. ddH_2_O served as the negative control. Marker, size marker. Right: chromatogram from Sanger sequencing of the PCR product, showing the reading frame at the break points. (**D**) *ACTB::TXNDC5*. Left: Graphical representation of the formation of *ACTB::TXNDC5* fusion at the chromosome level. Middle: RT-PCR of *ACTB::TXNDC5* from BM cells and oral epithelial cells. ddH_2_O served as the negative control. Marker, size marker. Right: chromatogram from Sanger sequencing of the PCR product, showing the reading frame at the break points.

To further validate the specificity of each fusion gene and exclude potential germline events, comparative analyses of BM and oral epithelial cell samples obtained from 22 patients were performed. A total of 159 fusion genes were subjected to further validation, of which 23 were successfully confirmed by RT-PCR and Sanger sequencing (Figure 3 and Figures S1–S3). Among them, four fusion genes, including *DDX5::EEF1A1*, *YAF2::RYBP*, *ARFIP1::PVT1*, and *ACTB::TXNDC5*, were detected in patients 1 through 4, respectively (Figure 3A–D). Simultaneously, two distinct sets of fusion genes were validated in BM samples from two different patients: the first set included *KDM7A::MKRN1*, *EDF1::RABL6*, *OAZ1::SGTA*, *OAZ1::TCF3*, *OAZ1::DAZAP1*, and *PTMA::CXCR4* (Figure S1A–F), while the second set comprised *YWHAE::KLF2*, *ELL::KLF2*, *FOSB::SF1*, *B2M::KLF2*, *ZNF292::PNRC1*, and *OAZ1::KLF16* (Figure S2A–F). Furthermore, additional fusion genes were validated in BM samples from five distinct patients, including the fusion *OAZ1::KLF2* (Figure S3A), a fusion pair (*HNRNPA2B1::EEF1A1* and *DDX5::SRSF7*) (Figure S3B,C), and three individual fusion genes (*OAZ1::ZBTB7A* [Figure S3D], *OAZ1::METRNL* [Figure S3E], and *PLXNB2::SCO2* [Figure S3F]).

Collectively, fusion genes were reliably detected and validated in 54.5% of patients. These results further indicate that fusion genes represent potential molecular markers in patients with MM.

### 3.5. Fusion Gene-Based MRD Monitoring Enables Early Detection of Relapse in Patients with MM

**Figure 4:**
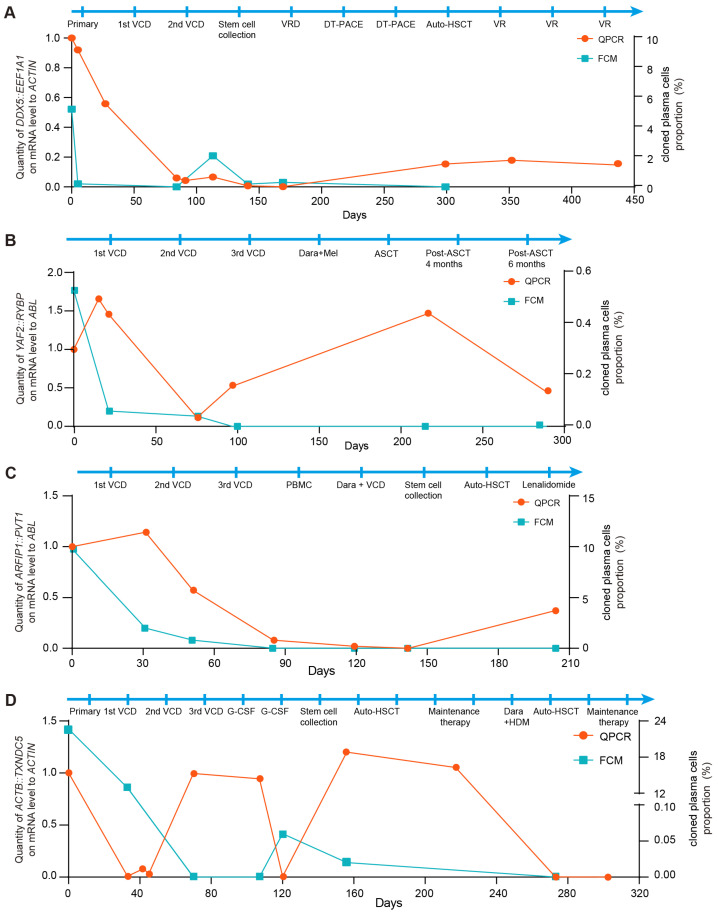
Fusion gene-based MRD monitoring enables early detection of relapse in patients with multiple myeloma. (**A**) Relative quantification of *DDX5::EEF1A1* fusion was performed using the fusion gene-based qPCR assay in case 1. *ACTIN* expression served as control. FCM analysis of the clonal plasma cell population in the BM from the immunophenotyping test report of case 1. (**B**) Relative quantification of *YAF2::RYBP* fusion was performed using the fusion gene-based qPCR assay in case 2. *ABL* expression served as control. FCM analysis of the clonal plasma cell population in the BM from the immunophenotyping test report of case 2. (**C**) Relative quantification of *ARFIP1::PVT1* fusion was performed using the fusion gene-based qPCR assay in case 3. *ABL* expression served as control. FCM analysis of the clonal plasma cell population in the BM from the immunophenotyping test report of case 3. (**D**) Relative quantification of *ACTB::TXNDC5* fusion was performed using the fusion gene-based qPCR assay in case 4. *ACTIN* expression served as control. FCM analysis of the clonal plasma cell population in the BM from the immunophenotyping test report of case 4.

As shown in Figure 4, the downward trends in FCM results and fusion gene-based molecular monitoring in BM were consistent from the initial disease stage through remission in patients with MM. In case 1, the fusion copy number increased despite negative FCM results following remission. Notably, a significant reduction in fusion gene copy number was observed after allogeneic hematopoietic stem cell transplantation (HSCT), and the fusion gene-based method provided an earlier indication of relapse compared to FCM (Figure 4A). In case 2, representing NSMM, an elevation in fusion gene copy number was detected despite negative FCM results after HSCT. Importantly, the presence of immature plasma cells identified by BM cytology corroborated these molecular findings (Figure 4B). In case 3, fusion transcripts were detectable in the patient’s BM using the fusion gene-based approach, whereas the FCM results remained negative. Meanwhile, the patient’s clinical status shifted from complete remission to partial remission according to BM cytology, further suggesting early relapse during maintenance therapy (Figure 4C). In case 4, both fusion gene and FCM signals decreased concurrently after the first transplantation. However, persistent fusion gene positivity suggested a potential relapse during maintenance treatment therapy. Based on prior evidence that MRD assessment can provide early relapse warnings, a second transplantation was performed, after which both markers became negative and the patient achieved a favorable prognosis (Figure 4D).

Collectively, these findings indicate that fusion gene-based MRD monitoring provides high analytical detectability for early relapse detection and may offer valuable guidance for clinical management.

## 4. Discussion

**Figure 5:**
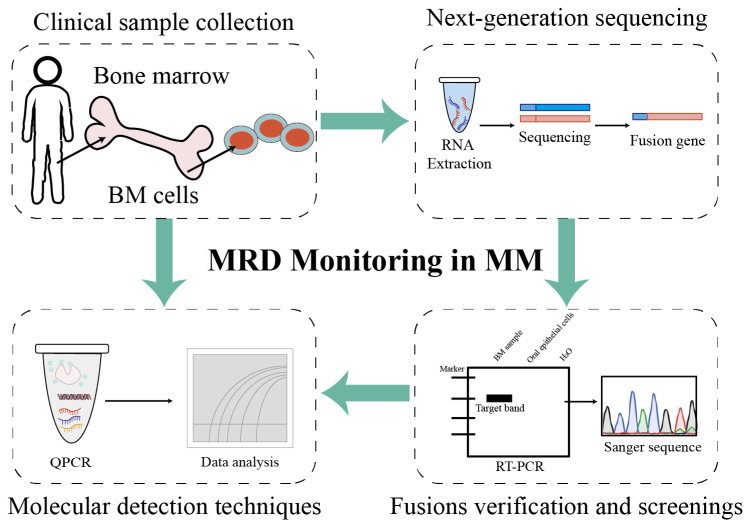
Project pipeline of fusion genes as biomarkers for MRD monitoring in patients with multiple myeloma. Firstly, bone marrow mononuclear cells were isolated via density gradient centrifugation, and plasma cells were isolated from bone marrow mononuclear cells using a commercial CD138-positive magnetic-activated cell sorting (MACS) kit. Then, the total RNAs of plasma cells were extracted using a commercial kit and were quality-controlled. Candidate fusion genes were identified through NGS-based transcriptome analysis (as detailed in the preceding Section 2), and positive fusion events were validated for specificity via nested RT-PCR or Sanger sequencing. Patient-specific primers/probes targeting validated fusion transcripts were designed (analytical detectability ≥ 10^−4^, equivalent to 1 positive cell per 10,000 normal cells), and reaction conditions for RT-qPCR or ddPCR were optimized to enable the reliable detection of low-abundance fusion transcripts. Follow-up samples were tested using the established assay to quantify fusion gene expression levels: MRD positivity was defined as detectable fusion transcript levels above the assay’s lower limit of detection (LOD), whereas MRD negativity indicated undetectable transcripts. Finally, MRD status (positive/negative) was correlated with clinical outcomes to guide treatment adjustments: intensified therapy was considered for patients with positive MRD and maintenance therapy for patients with positive MRD, in accordance with clinical guidelines.

This study provides a comprehensive overview of the fusion transcript landscape associated with chromosomal translocations in MM using an RNA-seq approach (Figure 5). Additionally, fusion events were compared across different chromosomes and associated biological processes. Through the validation and detection of patient-specific fusion genes in patients with MM, a standardized and individualized monitoring workflow tailored to each patient was established. Building upon previous research, the dataset and analytical strategies generated in this study are expected to serve as valuable resources for future investigations and to facilitate the clinical translation of fusion gene-based biomarkers.

The analysis revealed an average of seven fusion events per patient (range: 0–24), which is highly consistent with the findings reported by Cleynen A et al. [16], who reported an average of 5.5 fusions per patient (range: 0–24) and an average of 6.1 fusions in MM cell lines. However, a key methodological distinction in transcript analysis exists between the two studies. The current study employed the NovaSeq platform with 150 bp paired-end reads, whereas Cleynen et al. applied the TopHat method to data of shorter read length (50/75 bp). This methodological difference suggests that, despite the convergent results, the underlying analytical detectability and specificity may be influenced by technical choices. It is particularly noteworthy that Kortüm KM et al. observed an even lower detection rate (average of 2.7 fusions per patient) using longer 200 bp reads (IonOneTouch system) [17]. Collectively, this implies that simply increasing read length is not the decisive factor in improving fusion detection efficiency; rather, the interaction among sequencing platform specificity, analytical algorithms, and read length likely plays a critical role in generating discrepancies across studies. Therefore, future research should move beyond standardized sequencing frameworks and systematically evaluate the combined impact of the “platform–read length–algorithm” methodological triad on fusion detection rates and accuracy. This approach will help distinguish true biological variation from technical artifacts and advance MM gene fusion detection toward clinically reproducible and comparable applications.

Regarding the characteristics of the fusion events, chromosomes 1 and 19 were more frequently involved in fusions. This observation may be explained by the fact that chromosome 1 is the largest human chromosome, containing 3141 genes and 991 pseudogenes [18], whereas chromosome 19 has the highest gene density among human chromosomes, with 1461 protein-coding genes and 321 pseudogenes [19]. Specifically, *OAZ1* and *KLF2* were confirmed to exhibit the highest recurrence frequencies at the left and right breakpoints, respectively, with both genes mapped to chromosome 19. *OAZ1*, a key negative regulator of polyamine metabolism, triggers the aberrant activation of the polyamine synthesis pathway in MM cells when dysregulated, resulting in significantly elevated polyamine levels that promote the uncontrolled proliferation of myeloma cells [20,21]. Additionally, *KLF2*, a Krüppel-like zinc-finger transcription factor, is highly expressed in early B-cell progenitors [22,23,24]. The KDM3A-KLF2-IRF4 axis regulates *ITGβ7*, which mediates MM cell adhesion and BM homing [25]. *KLF2* further modulates the angiogenic factors *EGFL7* and *ITGβ3*, thereby promoting MM cell expansion [26]. Specifically, the enrichment analysis revealed that the target gene set was significantly enriched for functions related to transcriptional regulation and mRNA metabolism. These genes are implicated in RNA splicing, transcription factor activity, and cell–microenvironment adhesion signaling. This observation aligns with the classic pathogenesis of MM; for instance, t (4;14) translocation leads to the overexpression of *FGFR3* and *MMSET*/*NSD2*, whereas t (14;16) translocation results in *c-MAF* overexpression, both of which are hallmark examples of transcriptional dysregulation [27]. Therefore, transcriptional dysregulation plays a critical role in promoting myelomagenesis and disease progression.

In this study, it was demonstrated that these novel fusion gene transcripts can be used to detect MRD at the transcriptional level. This method demonstrated higher detectability than FCM-based techniques, which is consistent with previous findings [28,29]. Ren Yuyue et al. demonstrated that the RNA-seq method based on NGS reads (targeted IGH-CDR3-DNA-NGS) detected IG tag sequences with favorable detection rates in both cell lines and MM samples [8]. The precise monitoring of non-immunoglobulin (non-IG) fusion genes was achieved by utilizing qPCR technology, which facilitated accurate relapse prediction and informed clinical therapeutic decisions. While NGS generally offers higher analytical detectability and does not require target-specific primer design in validated assays, its higher cost and longer turnaround time render it more suitable for exploratory studies aimed at novel fusion discovery [29]. On the other hand, RT-qPCR offers advantages such as high reproducibility, rapid detection, and relatively low cost, making it well suited for the rapid validation and clinical monitoring of known fusion events [11]. Therefore, in the detection of gene fusions in multiple myeloma, these two methods can serve complementary roles: RNA-seq is ideal for comprehensive screening and discovery, while RT-qPCR is better suited for subsequent longitudinal monitoring and clinical application. Furthermore, a fusion gene-based MRD monitoring platform for acute lymphoblastic leukemia was established in a previous study and yielded positive clinical feedback [30]. Building on this foundation, the platform was successfully extended to establish a non-IG fusion gene-based molecular monitoring system for MM. This system has enabled the precise tracking of disease progression and guided personalized treatment strategies.

## 5. Conclusions

In summary, NGS determined that each patient with MM harbored at least one fusion gene, which can serve as a molecular marker for MRD monitoring. However, the involvement of these fusion genes in the initiation and progression of MM was not investigated. Future studies will expand the patient cohort to identify additional recurrent fusion genes, elucidate their pathogenic mechanisms, and establish standardized protocols for the clinical application of fusion genes in MRD monitoring of patients with MM.

## Figures and Tables

**Table 1:** Primer sequences used for PCR and QPCR.

Primer	Sequence (5′-3′)
*DDX5::EEF1A1*-F	ATGTCGGGTTATTC
*DDX5::EEF1A1*-R	TTCAATGGTTCTTTTGTCGATGCCA
*YAF2::RYBP*-F	ATGGGAGACAAGAAGAGCCCCACCAGGC
*YAF2::RYBP*-R	AGGTGCCTTTCCTCACATCGCAGATGCT
*ARFIP1::PVT1*-F	TTACCCTAAGAAAGCCAGTC
*ARFIP1::PVT1*-R	TGGGCAGGGTAGAT
*ACTB::TXNDC5*-F	GAGCACAGAGCCTCGCCTTTGCCGATCC
*ACTB::TXNDC5*-R	GCTGTTGTATTTGTCTCCCAGGTCATTCCA
*ABL*-F	TCGAGCAGGAGATGGCCACTGCCGCATC
*ABL*-R	GACTGTTGACTGGCGTGAT
*ACTIN*-F	GAGCGCGGCTACAGCTT
*ACTIN*-R	TCCTTAATGTCACGCACGATTT

**Table 2:** Patient cohort description and demographics.

Characteristics	Multiple Myeloma (*N* = 22)
Sex (*N*, %)	
Male	15 (68.2%)
Female	7 (31.8%)
Age (years)	
Median (range)	57 (42–72)
Stage (*N*, %)	
DS	
I	1 (4.5%)
II	3 (13.6%)
III	18 (81.8%)
ISS	
I	5 (22.7%)
II	4 (18.2%)
III	13 (59.1%)
R-ISS	
I	3 (13.6%)
II	7 (31.8%)
III	12 (54.5%)
Immunophenotype (*N*, %)	
IgG	9 (40.9%)
IgA	5 (22.7%)
IgD	1 (4.5%)
Light chain	5 (22.7%)
Biclonal	1 (4.5%)
Non-Secretory	1 (4.5%)
Light Chain (*N*, %)	
K	13 (59.1%)
L	8 (36.4%)

DS = Durie–Salmon Staging System, ISS = International Staging System, R-ISS = Revised International Staging System.

## Data Availability

The original contributions presented in this study are included in the article/Supplementary Materials. Further inquiries can be directed to the corresponding authors.

## Supplementary Materials

The following supporting information can be downloaded at: https://www.mdpi.com/article/10.3390/biomedicines14020354/s1, Figure S1. Validation of fusion gene specificity and identification of potential molecular markers in patients 5 and 6. Figure S2. Validation of fusion gene specificity and identification of potential molecular markers in patients 7 and 8. Figure S3. Validation of fusion gene specificity and identification of potential molecular markers from samples from multiple patients. Table S1. Primer sequences used for PCR.

## Abbreviations

MM	Multiple myeloma
IGH	Immunoglobulin heavy chain
NSMM	Non-secretory multiple myeloma
MRD	Minimal residual disease
BM	Bone marrow
FCM	Flow cytometry
PCR	Polymerase chain reaction
RT-qPCR	Reverse transcription quantitative polymerase chain reaction
NGS	Next-generation sequencing
HSCT	Hematopoietic stem cell transplantation
