# Supplementary material for: Integrated RNA-seq and RT-qPCR Workflow Identifies Non-IGH Fusion Transcripts as Individualized Molecular Markers for Monitoring Multiple Myeloma
Source: Biomedicines. 2026 Feb 3;14(2):354. doi: 10.3390/biomedicines14020354 (PMC12937900; doi:10.3390/biomedicines14020354)
Supplement: Supplementary file 1 [file biomedicines-14-00354-s001.zip › Supplementary Figure.pdf]

Supplementary Figure

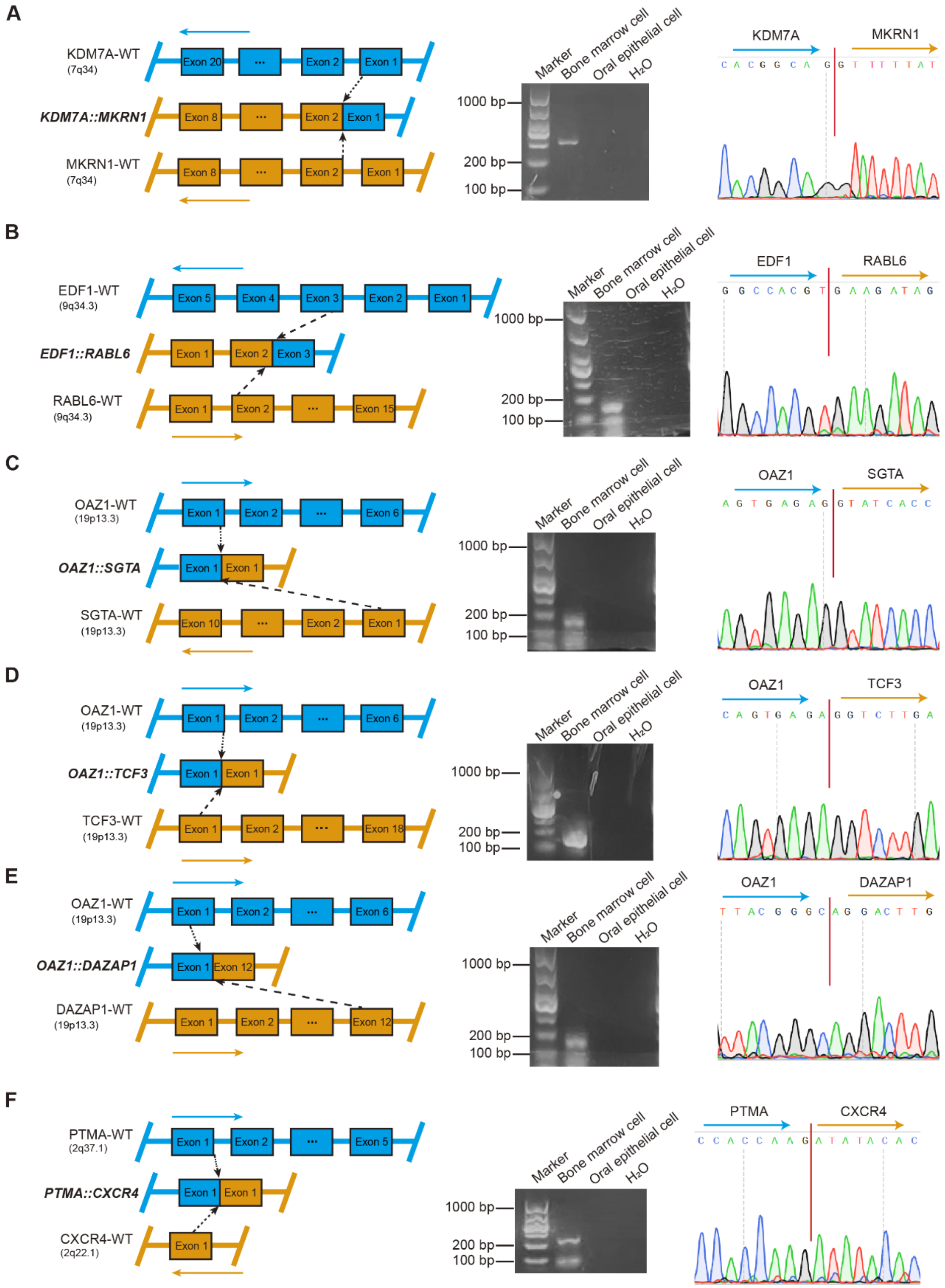

Figure S1. Validation of fusion gene specificity and identification of potential molecular markers in patients 5 and 6. A-E: *KDM7A::MKRN1*, *EDF1::RABL6*, *OAZ1::SGTA*, *OAZ1::TCF3*, and *OAZ1::DAZAP1* are detected in patient 5. Left: Graphical representation of the formation of fusion genes at the chromosome level. Middle: RT-PCR of fusion genes from BM cells and oral epithelial cells. ddH_2O served as the negative control. Marker, size marker; Right: chromatogram from Sanger sequencing of the PCR product, showing the reading frame at the break points. **F:** *PTMA::CXCR4* is detected in patient

6. Left: Graphical representation of the formation of fusion genes at the chromosome level. Middle: RT-PCR of fusion genes from BM cells and oral epithelial cells. ddH_2O served as the negative control. Marker, size marker; Right: chromatogram from Sanger sequencing of the PCR product, showing the reading frame at the break points.

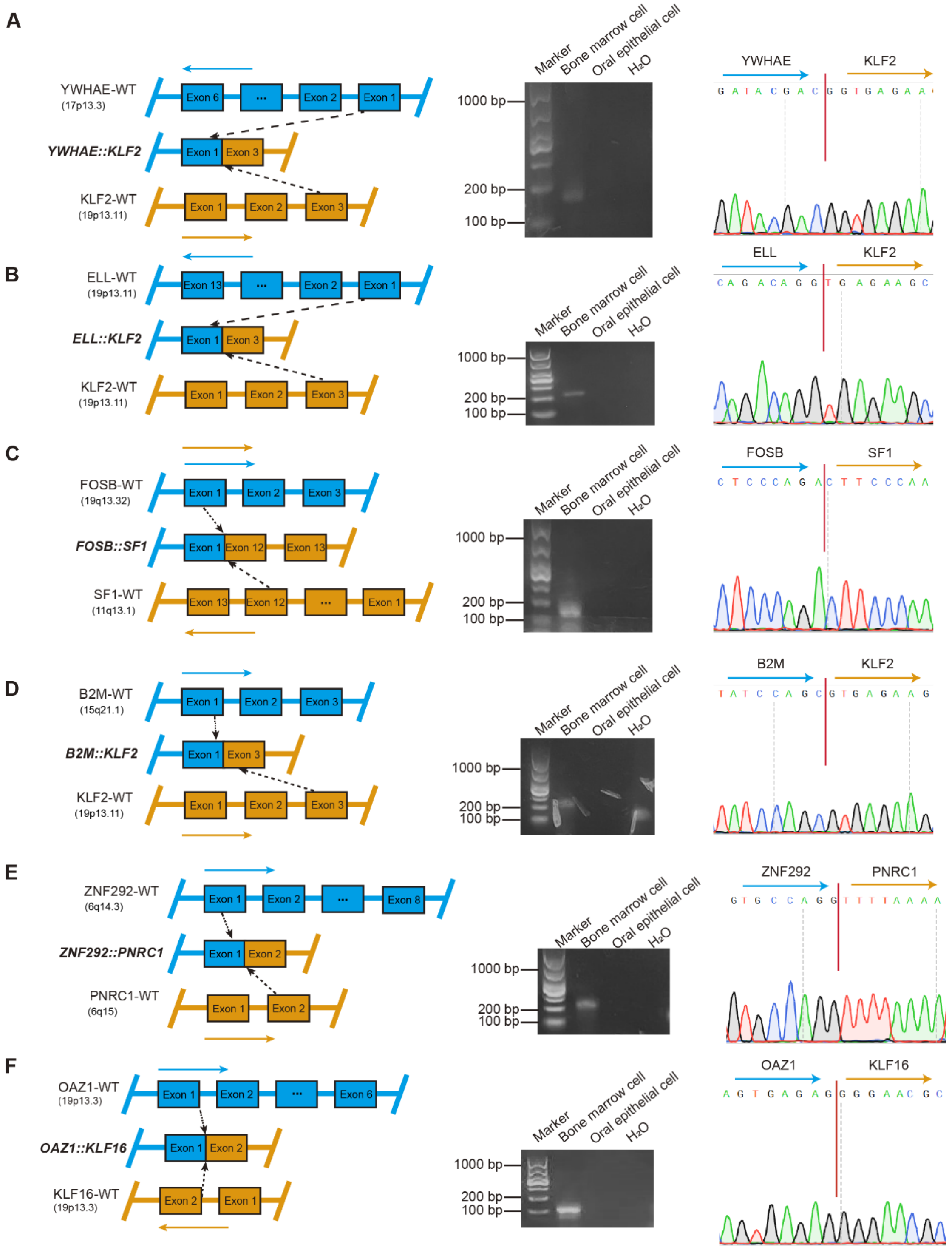

Figure S2. Validation of fusion gene specificity and identification of potential molecular markers in patients 7 and 8. A-E: *YWHAE::KLF2*, *ELL::KLF2*, *FOSB::SF1*, *B2M::KLF2*, and *ZNF292::PNRC*1 are detected in patient 7. Left: Graphical representation of the formation of fusion genes at the

chromosome level. Middle: RT-PCR of fusion genes from BM cells and oral epithelial cells. ddH_2O served as the negative control. Marker, size marker; Right: chromatogram from Sanger sequencing of the PCR product, showing the reading frame at the break points. **F:** *OAZ1::KLF16* is detected in patient 8. Left: Graphical representation of the formation of fusion genes at the chromosome level. Middle: RT-PCR of fusion genes from BM cells and oral epithelial cells. ddH_2O served as the negative control. Marker, size marker; Right: chromatogram from Sanger sequencing of the PCR product, showing the reading frame at the breakpoints.

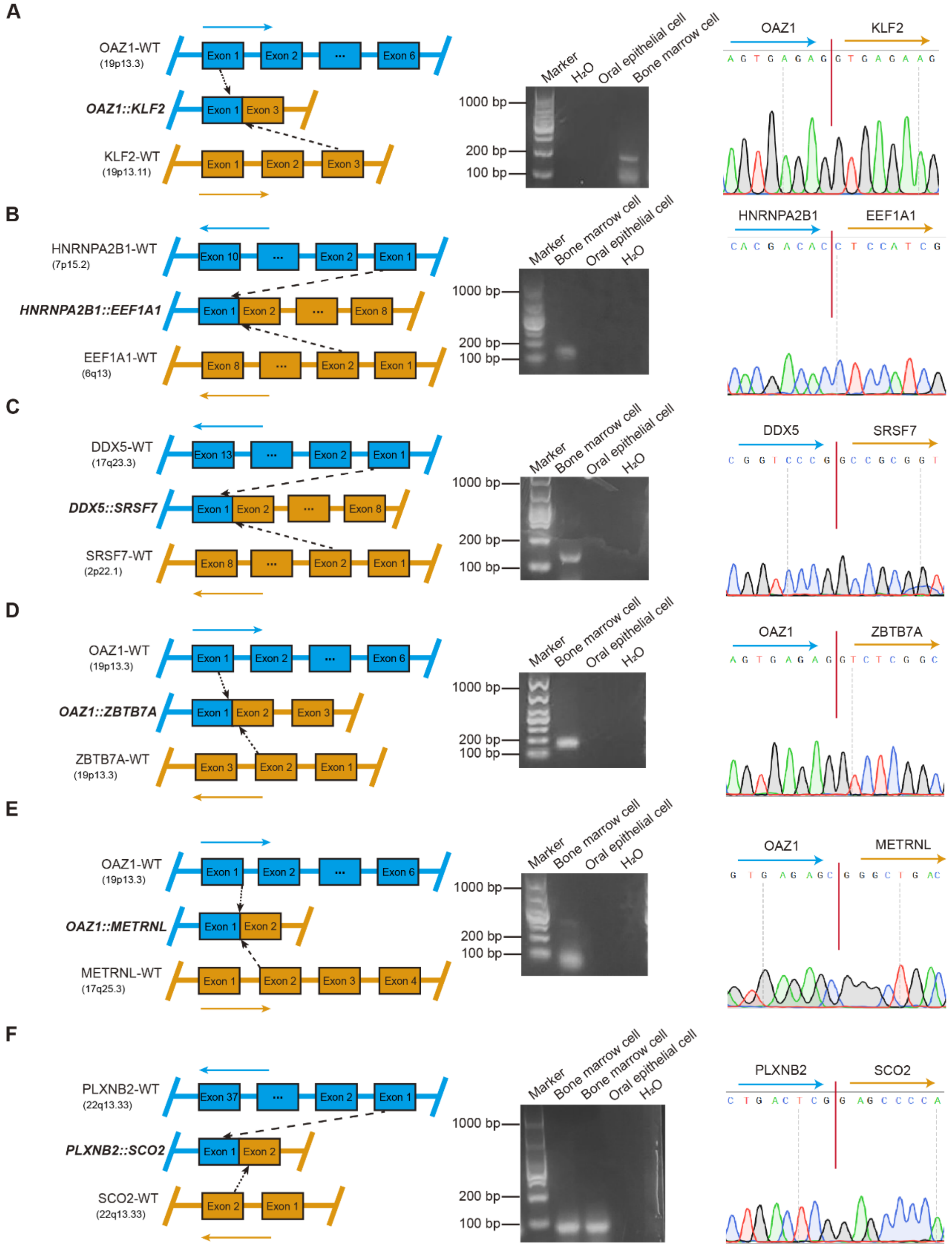

Figure S3. Validation of fusion gene specificity and identification of potential molecular markers from samples from multiple patients. A-F: *OAZ1::KLF2* **(A)**, *HNRNPA2B1::EEF1A1* **(B)** and *DDX5::SRSF7* **(C)**, *OAZ1::ZBTB7A* **(D)**, *OAZ1::METRNL* **(E)**, and *PLXNB2::SCO2* **(F)** were detected in patient 9-14, respectively. Left: Graphical representation of the formation of fusion genes at the chromosome level. Middle: RT-PCR of fusion genes from BM cells and oral epithelial cells. ddH_2O served as the negative control. Marker, size marker; Right: chromatogram from Sanger sequencing of the PCR product, showing the reading frame at the breakpoints.
