# Supplementary material for: Integrated RNA-seq and RT-qPCR Workflow Identifies Non-IGH Fusion Transcripts as Individualized Molecular Markers for Monitoring Multiple Myeloma
Source: Biomedicines. 2026 Feb 3;14(2):354. doi: 10.3390/biomedicines14020354 (PMC12937900; doi:10.3390/biomedicines14020354)
Supplement: Supplementary file 1 [file biomedicines-14-00354-s001.zip › Supplementary Table.pdf]

Supplementary Table S1. Primer sequences used for PCR.

Primer	Sequence (5′-3′)
KDM7A::MKRN1-F	CCACGGCAGGTATTTTATGC
KDM7A::MKRN1-R	TGAACCTGCTCCTACAGTTG
EDF1::RABL6-F	CTGGCCAGAACAAACAACAT
EDF1::RABL6-R	CCCCGGATCACTATCTTCAC
OAZ1::SGTA-F	TGCAGCGGATCCTCAATAG
OAZ1::SGTA-R	GAGCCCAGAAGAGGTGATAC
OAZ1::TCF3-F	AGCAGTAGAGAGGTCTTGAG
OAZ1::TCF3-R	TCCCAGGAATGTGGATGAAG
OAZ1::DAZAP1-F	TGCAGCGGATCCTCAATAG
OAZ1::DAZAP1-R	CTCAAGTCCTGCCCGTAAC
PTMA::CXCR4-F	CTCCGAAATCACCA
PTMA::CXCR4-R	TTCTTCTGGTAACCCATGACCAGGA
YWHAE::KLF2-F	GAGCAGGCTGAGCGATAC
YWHAE::KLF2-R	CGCGAGAAGGCACGAT
ELL::KLF2-F	TTAGCGACGGCAGCAA
ELL::KLF2-R	CAGATGGCACTGGAATGG
FOSB::SF1-F	ATGTTTCAGGCTTTCCC
FOSB::SF1-R	TCCTCTTCCGCTTCTTAC
B2M::KLF2-F	GGCCTTAGCTGTGCTCG
B2M::KLF2-R	TGTGCCGTTTCATGTGC
ZNF292::PNRC1-F	GAGCGGAGGTTGAGTTG
ZNF292::PNRC1-R	AACCAAGATCACCTCTGCAA
OAZ1::KLF16-F	TGCAGCGGATCCTCAATAG
OAZ1::KLF16-R	CTGCCAGTCACAAGCAAAAG
OAZ1::KLF2-F	ATGCCGCTCCTAAG
OAZ1::KLF2-R	ACTGCAACTGG
HNRNPA2B1::EEF1A1-F	GCTGAAGCGACTGA
HNRNPA2B1::EEF1A1-R	CTTGCCCGAATCTA
DDX5::SRSF7-F	CGAGTGACCGAGAC
DDX5::SRSF7-R	CCACAAAGGCAAAT
OAZ1::ZBTB7A-F	AATAGCCACTGCTTCGC
OAZ1::ZBTB7A-R	GCTCGTTCAGCCCACT
OAZ1::METRNL-F	CACCATGCCGCTCCTAAG
OAZ1::METRNL-R	CGCACAGCGCAGATACAC
PLXNB2::SCO2-F	ACAAAGGCCCCGTCT
PLXNB2::SCO2-R	TCTCCAGCTCGTCTGGGCAGATGTC
